# Clinical study of autoantibodies in type 1 diabetes mellitus children with ketoacidosis or microalbuminuria

**DOI:** 10.1002/jcla.24164

**Published:** 2021-12-03

**Authors:** Mingying Zhang, Xinhui Wang, Rui Wang, Jianbo Shu, Xiufang Zhi, Chunyu Gu, Linjie Pu, Chunquan Cai, Wei Yang, Ling Lv

**Affiliations:** ^1^ Department of Pediatric Endocrinology Tianjin Children's Hospital (Tianjin University Children's Hospital) Tianjin China; ^2^ Graduate College of Tianjin Medical University Tianjin China; ^3^ Institute of Pediatric (Tianjin Key Laboratory of Birth Defects for Prevention and Treatment) Tianjin Children's Hospital (Tianjin University Children's Hospital) Tianjin China; ^4^ Department of Pediatric Neurosurgery Tianjin Children's Hospital (Tianjin University Children's Hospital) Tianjin China; ^5^ Tianjin Medical Device Evaluation and Inspection Center Tianjin China

**Keywords:** autoantibody, children, diabetic ketoacidosis, microalbuminuria, type 1 diabetes mellitus, zinc transporter‐8 autoantibodies

## Abstract

**Aims:**

The study aimed to investigate the value of autoantibodies in predicting the risk of ketoacidosis or microalbuminuria in children with type 1 diabetes mellitus.

**Methods:**

Clinical data and laboratory indicators of 80 patients with type 1 diabetes admitted to the Department of Endocrinology in Tianjin Children's Hospital, from June 2017 to March 2019, were retrospectively analyzed. The patients were divided into two groups: diabetes without ketoacidosis group (*n* = 20) and diabetes with ketoacidosis group (*n* = 60). The differences in general data, laboratory test indexes, and autoantibodies between the two groups were analyzed. Finally, ROC curves and multivariate logistic regression analysis were used to explore the value of autoantibodies in patients with ketoacidosis or microalbuminuria.

**Results:**

A total of 80 children with type 1 diabetes were assessed, including 35 boys and 45 girls, ranging in age from 10 months to 15 years. The concentration of GADA, IA2A, and ZnT8A was not statistically different between the two groups, but the positive rate of ZnT8A was statistically significant (*p* = 0.038) and had a diagnostic value for the occurrence of ketoacidosis (*p* = 0.025). ZnT8A‐positive patients had a higher titer of IA2A and a more frequent prevalence of GADA and IA2A than ZnT8A‐negative patients (*p* < 0.01). In multivariate logistic regression analyses, the presence of positive ZnT8A was associated with a higher risk of microalbuminuria independent of age, sex, and BMI (OR = 4.184 [95% CI 1.034~16.934], *p* = 0.045).

**Conclusions:**

The positive ZnT8A had diagnostic value for ketoacidosis in children with type 1 diabetes and had the highest specificity among the three kinds of autoantibodies. Moreover, ZnT8A positivity was related to a higher titer of IA2A and more frequent occurrence of multiple diabetes‐related autoantibodies. Besides, the presence of positive ZnT8A was an independent risk factor of microalbuminuria in children with type 1 diabetes. Therefore, we can infer that positive ZnT8A may be related to ketoacidosis and microalbuminuria, accelerating the progression of T1DM.

## Graphical abstract

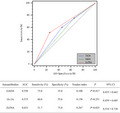

## 1 INTRODUCTION

Type 1 diabetes mellitus (T1DM), characterized by insulin deficiency and resultant hyperglycemia, results from immune dysregulation in which the immune response is specifically directed against pancreatic β‐cells.^1^ The mechanism of this process remains unclear and T1DM is considered a multifactorial disease.^2^ To this point, no single factor inducing the destructive process has been identified. Genetic susceptibility and many environmental agents play a role in disease emergence.^3^ T1DM accounts for about 90% of all types of diabetes in childhood, with an average annual increase of 5% to 34% in the incidence of children younger than 5 years old.^4^ Diabetic ketoacidosis (DKA) is the most common cause of death in children and young adults with T1DM characterized by metabolic acidosis, hyperketonemia, and hyperglycemia.^5^ The symptoms of DKA can appear within a few hours, including nausea, vomiting, polyuria, excessive thirst, and cerebral edema. Because of the high incidence rate and sequela of DKA, the prevention of DKA is still a challenge for T1DM patients. As the first step to prevent DKA, it is necessary to identify people with an increased risk of DKA in time, and the level of awareness of diabetic symptoms among healthcare professionals seems to be the key for DKA prevention.

Diabetic nephropathy (DN), a major microvascular complication of both T1DM and T2DM, is the leading cause of the end‐stage renal disease (ESRD) worldwide.^6^ The lifetime risk of DN in T1DM has traditionally been estimated at ~50% but may exceed 70%.^7^ The initial stage of DN is characterized by the development of microalbuminuria (MA) and hyperfiltration.^8^ The traditional concept of DN holds that MA (albumin excretion rate >30 mg/24 h) is the fundamental early prognostic indicator that heralds macroalbuminuria, indicating a need for additional treatments.^7^ In light of the dynamic process of T1DM, there is a need to identify risk factors associated with MA of kidney disease.

In the natural course of the disease, although T1DM is mainly caused by T lymphocyte–mediated destruction of insulin‐producing β cells within pancreatic islets, detection of islet autoantibodies in peripheral blood is currently the most reliable marker for detecting the autoimmune process leading to clinical T1DM.^9^ The presence of autoantibodies at a young age, high autoantibody titer, and high expression of autoantibodies are clear risk indicators for T1DM.^10^ We routinely detect autoantibodies including autoantibodies to insulin (IAA), glutamic acid decarboxylase autoantibodies (GADA), protein tyrosine phosphatase autoantibodies (IA2A), and zinc transporter‐8 autoantibodies (ZnT8A). The earliest development of autoantibody was found in children with single IAA, with a sharp peak in incidence observed at age 9 months.^11^ Islet autoantibodies usually appeared years before overt clinical disease and nearly all children who have progressed to T1DM usually expressed two or more autoantibodies.^12^ In the TEDDY study, higher IAA and IA2A levels will increase the risk of T1DM in children.^13^ ZnT8 is highly expressed in pancreatic beta cells, where zinc plays an important physiological role and is essential for normal insulin storage.^14^ ZnT8A has been used as an independent predictor of diabetes risk and has been included in the risk prediction and prevention study of T1DM.^15^

In the current literature, there were fewer studies on the clinical correlation of autoantibodies in T1DM children with DKA or MA. This study analyzed the clinical related factors and autoantibodies of 80 children with T1DM. The purpose of this study was to explore the diagnostic value of autoantibodies in predicting the risk of DKA and investigate risk factors of MA.

## 2 MATERIALS AND METHODS

### 2.1 Patients

One hundred and twenty children with the first‐onset T1DM were hospitalized in the Department of Endocrinology at Tianjin Children's Hospital from June 2017 to March 2019, excluding those with chronic diseases such as heart, brain, liver, kidney, or co‐infectious diseases, and 80 children (boys:35; girls:45) were finally selected. The age of the patients ranged from 10 months to 15 years, with a median age of 4 years and 4 months. All patients were given reasonable insulin replacement therapy. The patients were divided into two groups: the T1DM group without DKA (20 cases) and the T1DM group with DKA (60 cases).

Informed consent documents were signed by all parents of the children included in the study after a detailed explanation of the study. The study was approved by the medical ethics committee of Tianjin children's hospital.

### 2.2 Diabetes diagnosis

Type 1 diabetes mellitus was diagnosed based upon WHO criteria, combined with related clinical symptoms, such as drink more, eat more, or lose weight.^16^ For those who met the WHO criteria but were asymptomatic, it was recommended to repeat the test on the following day to confirm the diagnosis.

The clinical manifestation of children with T1DM was acute onset, which could be accompanied by DKA; C‐peptide was lower than normal or lower detection limit at the beginning of disease; it may be positive for GADA, IA2A, or ZnT8A; no acanthosis nigricans; it can be associated with a family history of diabetes and other autoimmune diseases, such as Graves's disease and Hashimoto's thyroiditis.

### 2.3 DKA diagnosis

The diagnostic criteria for DKA, as defined by the American Diabetes Association, are hyperglycemia, defined as a serum glucose level greater than 200 mg/dl or about 13.9 mmol/L; venous pH <7.3 or serum bicarbonate <15 mmol/L; ketonemia greater than 31 mg/dl or ketonuria greater than 80 mg/dl.

DKA is classified as mild if the patient's pH is <7.3 and serum bicarbonate is <15 mmol/L; moderate if the venous pH is <7.2 and the bicarbonate is <10 mmol/L; and severe if the venous pH is <7.1 and bicarbonate is <5 mmol/L.

### 2.4 Clinical and laboratory data

General physical examination and standard laboratory test were performed on every patient on admission. Bodyweight and height were measured in a standardized way, and body mass index (BMI, weight in kg /square of height in meters) was calculated. Blood samples from participants were collected between 7 and 9 a.m. following an overnight fast. The plasma was separated, aliquoted, and stored at −80°C before use. Plasma glucose, HbA1c, C‐peptide, C‐reactive protein (CRP), procalcitonin (PCT), MA, triiodothyronine (T3), thyroxine (T4), and thyroid‐stimulating hormone (TSH) levels were measured at the diagnostic laboratory of Tianjin Children's hospital.

The first morning urine was collected from a tube of 20 mL, centrifuged at 3500 r/min for 10 min, and the supernatant was taken to detect urinary MA. Urinary MA was determined by immune scattering turbidimetry with Ilicon reagent and Hitachi 7600 automatic biochemical analyzer. We defined the levels of MA <30 mg/L for T1DM patients without MA, and MA ≥30 mg/L for T1DM patients with MA.

### 2.5 Autoantibody assays

To confirm autoimmune diabetes origin, three kinds of typical autoantibodies were tested. We applied a commercially available enzyme‐linked immunosorbent assay to measure diabetes‐related autoantibodies. GADA, IA2A, and ZnT8A were measured by ELISA kits from RSR^®^ Ltd, UK, based on the international standard substance NIBSC, which has passed the 2015 International diabetes‐related antibody standardized test verification (IASP 2015). All autoantibodies levels were calculated from a standard curve with concentration/dilution at the horizontal axis and background value 450 at the vertical axis, reported as μ/ml. The level of GADA was determined by the GADA ELISA Kit, GADA assay (positivity: >5 μ/ml). IA2A and ZnT8A were measured by the enzyme‐linked immunosorbent assay ([ElisaRSR™ IA‐2 Ab version 2, UK]/[ElisaRSR^TM^ ZnT8 Ab™, UK]). Autoantibody for positivity was >7.5 μ/ml and >15 μ/ml for IA2A and ZnT8A, respectively. The levels of GADA, IA2A, and ZnT8A were logarithmically transformed before analysis due to non‐normal distributions.

### 2.6 Statistical analysis

Continuous variables were presented as mean ± SD or median in the case of normal or non‐normal distribution, and differences between the two groups were examined by independent‐sample *t* test or Mann‐Whitney *U* test correspondingly. Categorical variables were described as counts (percentages) and compared by Pearson chi‐square test (Pearson *χ* ^2^ test) or Fisher's exact test appropriately. Receiver operating characteristic (ROC) curve analysis was used to explore the diagnostic value of autoantibodies in predicting the risk of DKA in T1DM children. Correlations between variables were assessed with Pearson or Spearman correlation. A multivariate logistic regression analysis was used to identify significant variables associated with MA. Statistical analyses were carried out using SPSS 22.0 software, and MedCalc version 19.0.7 was used to draw ROC curves. *p* < 0.05 was considered indicative of statistical significance.

## 3 RESULTS

### 3.1 Comparisons of clinical features between the two groups of children with T1DM

The age of patients without DKA was 8.42 ± 3.53 years and 7.13 ± 3.57 years in patients with DKA. The BMI was 15.97 ± 1.36 and 15.56 ± 2.19 kg/m^2^ between non‐DKA and DKA patients, respectively. There was no difference in age of onset, gender, and BMI (*p* > 0.05). T1DM patients with DKA had longer duration of hospitalization compared with those without (*p* < 0.001).

Plasma glucose, HbA1c, PCT, and MA levels in patients with DKA were all higher than that of patients without DKA (all *p* < 0.001). In contrast, T3 level and T4 level were lower in patients with DKA (all *p* < 0.001). There was no significant difference in C‐peptide, CRP, and TSH levels between the two groups (*p* > 0.05).

Comparisons of clinical features between the two groups of children with T1DM were summarized in Table 1.

**TABLE 1:** Comparisons of clinical features between the two groups of children with T1DM

	T1DM without DKA (*n* = 20)	T1DM with DKA (*n* = 60)	*p* value
Age of onset (year)	4.50 ± 1.66	4.26 ± 2.22	0.611
Age (year)	8.42 ± 3.53	7.13 ± 3.57	0.049
Male (%)	10/20 (50.0)	25/60 (41.7)	
Female (%)	10/20 (50.0)	35/60 (58.3)	0.515
BMI (kg/m^2^)	15.97 ± 1.36	15.56 ± 2.19	0.465
Duration (day)	5.40 ± 3.29	11.42 ± 4.72	<0.001
Glycemia (mmol/L)	11.28 ± 6.34	23.41 ± 9.15	<0.001
HbA1c (%) (mmol/mol)	9.03 ± 2.37	11.70 ± 2.17	<0.001
C‐peptide (nmol/L)	0.78 ± 1.88	0.15 ± 0.09	0.108
CRP (mg/L)	0.50 (0.10, 1.90)	1.00 (0.20, 4.05)	0.084
PCT (μg/mL)	0.05 (0.04, 0.07)	0.08 (0.05, 0.22)	<0.001
Microalbuminuria (mg/L)	2.40 (1.10, 6.85)	13.80 (3.50, 39.10)	<0.001
T3 (nmol/L)	1.65 ± 0.37	1.02 ± 0.45	<0.001
T4 (nmol/L)	107.61 ± 19.90	80.52 ± 36.57	<0.001
TSH (mIU/L)	1.85 ± 0.95	1.89 ± 1.90	0.893

### 3.2 Comparisons of three kinds of typical autoantibodies between the two groups of children with T1DM

There was no statistically significant difference in the levels of three kinds of autoantibodies between the two groups (*p* > 0.05), whereas the positive rate of ZnT8A was significantly higher in DKA patients compared with non‐DKA patients (51.7% vs. 25.0%, *p* = 0.038). The positive rate and titer of diabetes‐associated autoantibodies are shown in Table 2.

**TABLE 2:** Comparisons of three kinds of typical autoantibodies between the two groups of children with T1DM

	T1DM without DKA (*n* = 20)	T1DM with DKA (*n* = 60)	*p* value
GADA (%)	13/20 (65.0)	45/60 (75.0)	0.386
Ig (GADA) (μ/ml)	1.16 ± 1.10	1.44 ± 0.94	0.268
IA2A (%)	9/20 (45.0)	36/60 (60.0)	0.242
Ig (IA2A) (μ/ml)	1.27 ± 0.90	1.63 ± 1.18	0.154
ZnT8A (%)	5/20 (25.0)	31/60 (51.7)	0.038
Ig (ZnT8A) (μ/ml)	0.99 ± 0.90	1.27 ± 0.93	0.104

### 3.3 ROC curves of GADA, IA2A, and ZnT8A predicting DKA in T1DM children

**FIGURE 1:**
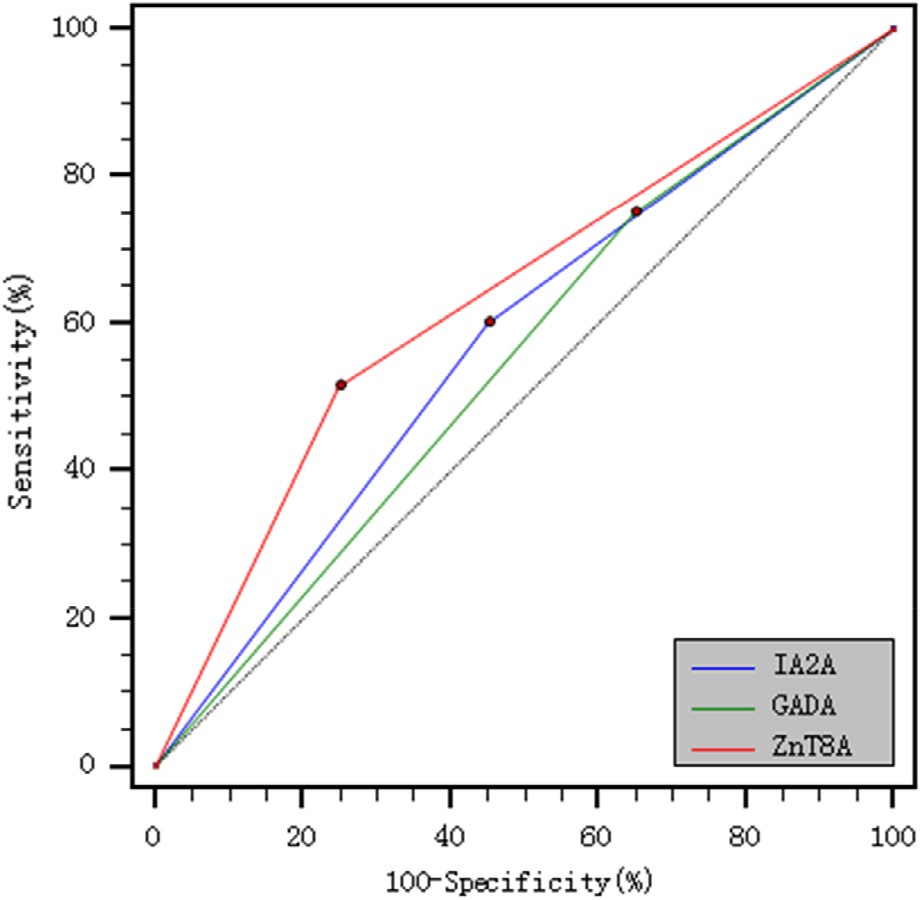
ROC curves of GADA, IA2A and ZnT8A predicting DKA in T1DM Children

ROC curves have shown the diagnostic value of ZnT8A positivity for T1DM children with DKA (*p* = 0.025). The area under the curve (AUC) of ZnT8A was the largest (0.633), with the highest Youden index (0.267), and the specificity was the best (75.0%); the AUC of GADA was the smallest (0.550), with the lowest Youden index (0.100), but the sensitivity was the best (75.0%). ROC curves of GADA, IA2A, and ZnT8A predicting DKA in T1DM children are shown in Figure 1.

### 3.4 Comparisons of T1DM children with positive and negative ZnT8A

Patients with positive ZnT8A had higher HbA1c levels and lower T3 levels than that of patients with negative ZnT8A (*p* = 0.024). There was no significant difference in glycemia, C‐peptide, T4, and TSH levels between the two groups (all *p* > 0.05). In the whole study subject, ZnT8A‐positive subjects were more prone to develop DKA (86.5% vs. 65.1%, *p* = 0.028). Besides, the risk of DKA in patients with positive ZnT8A was 3.207 times compared with patients with negative ZnT8A [OR = 3.207 (95% CI 1.034~9.944), *p* = 0.044]. ZnT8A‐positive subjects had a higher titer of IA2A and prevalence of GADA and IA2A (*p* < 0.01). However, there was no significant difference in the prevalence of thyroid disease (32.4% vs. 27.9%), family history of thyroid disease (8.1% vs. 2.3%), family history of diabetes (40.5% vs. 30.2%), and peripheral neuropathy (18.9% vs. 14.0%) between the two groups (all *p* > 0.05). Comparisons of T1DM children with positive and negative ZnT8A are shown in Table 3.

**TABLE 3:** Comparisons of T1DM children with positive and negative ZnT8A

	Positive ZnT8A (*n* = 37)	Negative ZnT8A (*n* = 43)	*p* value
Age of onset (year)	4.50 ± 1.66	4.26 ± 2.22	0.611
Age (year)	8.42 ± 3.53	7.13 ± 3.57	0.049
Sex (F:M)	19:18	26:17	0.413
BMI (kg/m^2^)	15.70 ± 2.12	15.61 ± 1.94	0.854
DKA (*n*) (%)	32 (86.5%)	28 (65.1%)	0.028
Glycemia (mmol/L)	21.66 ± 10.23	18.90 ± 10.22	0.232
HbA1c (%)	11.93 ± 2.34	10.55 ± 2.90	0.024
C‐peptide (pmol/ml)	0.15 ± 0.12	0.14 ± 0.17	0.756
CRP (mg/L)	0.80 (0.20, 2.00)	0.95 (0.20, 3.17)	0.727
PCT (μg/mL)	0.06 (0.04, 0.09)	0.07 (0.04, 0.17)	0.258
Microalbuminuria (mg/L)	6.10 (1.70, 19.02)	12.60 (3.27, 37.20)	0.041
T3 (nmol/L)	1.05 ± 0.52	1.35 ± 0.52	0.013
T4 (nmol/L)	86.22 ± 33.29	95.31 ± 33.08	0.182
TSH (mIU/L)	1.89 ± 2.18	1.76 ± 1.01	0.718
GADA (%)	86.5%	60.5%	0.009
Ig (GADA) (μ/ml)	1.56 ± 0.76	1.21 ± 1.12	0.105
IA2A (%)	73.0%	44.2%	0.009
Ig (IA2A) (μ/ml)	1.95 ± 1.12	1.91 ± 1.01	0.002
Thyroid diseases			
No (%)	25/37 (67.6)	31/43 (72.1)	0.660
Yes (%)	12/37 (32.4)	12/43 (27.9)	
Family history of thyroid diseases			
No (%)	34/37 (91.9)	42/43 (97.7)	0.504
Yes (%)	3/37 (8.1)	1/4 3 (2.3)	
Family history of diabetes			
No (%)	22/37 (59.5)	30/43 (69.8)	0.335
Yes (%)	15/37 (40.5)	13/43 (30.2)	
Peripheral neuropathy			
No (%)	30/37 (81.1)	37/42 (86.0)	0.548
Yes (%)	7/37 (18.9)	6/42 (14.0)	

### 3.5 Correlation analyses of MA levels and clinical measures

Pearson or Spearman correlation analyses shown that MA levels were positive correlated with age (*r* = 0.033, *p* = 0.703), glycemia (*r* = 0.270, *p* = 0.001), HbA1c (*r* = 0.375, *p* < 0.001), CRP (*r* = 0.082, *p* = 0.337), and PCT (*r* = 0.214, *p* = 0.011). MA levels were inversely correlated with BMI (*r* = −0.106, *p* = 0.274) and C‐peptide (*r* = −0.062, *p* = 0.474) in T1DM patients. Correlation analyses of MA levels and clinical measures are shown in Table 4.

**TABLE 4:** Correlation analysis of MA levels and clinical measures

Characteristics	MA (mg/L)	
	*r*	*p* value
Age (months)	0.033	0.703
BMI (kg/m^2^)	−0.106	0.274
Glycemia (mmol/L)	0.270	0.001
HbA1c (%)	0.375	<0.001
C‐peptide (nmol/L)	−0.062	0.474
CRP (mg/L)	0.082	0.337
PCT (ng/ml)	0.214	0.011

In multivariate logistic regression analyses, the presence of positive ZnT8A was associated with a higher risk of MA with/without adjusting for DKA status (*p* < 0.05). Multivariate logistic regression analyses are shown in Table 5.

**TABLE 5:** Predictors generated by multivariate logistic regression with MA levels as dependent variables with/without adjusting for DKA status

	Unadjusted			Adjusted		
	OR	95% CI	*p* value	OR	95% CI	*p* value
Age	0.853	0.632~1.152	0.300	0.959	0.681~1.352	0.812
Sex	0.992	0.270~3.651	0.991	0.728	0.133~4.000	0.715
BMI	0.715	0.494~1.034	0.075	0.733	0.474~1.133	0.162
Glycemia	1.054	0.989~1.122	0.104	1.035	0.944~1.135	0.460
HbA1c	1.134	0.926~1.388	0.225	1.025	0.941~1.117	0.569
C‐peptide	0.089	0.000~28.162	0.410	0.001	0.000~15.661	0.159
CRP	0.978	0.801~1.193	0.825	0.996	0.840~1.182	0.967
PCT	0.381	0.066~2.209	0.282	0.122	0.008~1.993	0.140
ZnT8A	0.239	0.059~0.968	0.045	0.147	0.024~0.909	0.039
(constant)			0.120			0.998

## 4 DISCUSSIONS

We confirmed that positive ZnT8A was more prevalent in T1DM children with DKA. Moreover, the diagnostic value of ZnT8A among three autoantibodies for T1DM children with DKA was the best in the specificity. The presence of positive ZnT8A was an independent risk factor of MA in children with T1DM.

DKA is a serious acute complication of T1DM. It had attracted more and more attention in recent years.^15^ The most common cause of death in T1DM patients is DKA. Even though much progress had been made in medical care in the past few decades, there were still many challenges in the management of this situation. In our study, we can conclude that the positive rate of ZnT8A has diagnostic value for T1DM with DKA in children. It is not entirely clear whether the onset age of diabetes affects the autoimmune status and clinical manifestations of patients with DKA.

The occurrence of autoantibodies (GADA, IA2A, or ZnT8A) is the sign of an autoimmune pathogenic apoptosis of β‐cells. More importantly, the prevalence of multiple types of autoantibodies identifies individuals at the highest risk for progression to T1DM.^17^ However, the prevalence of spontaneous IAA is low in Chinese patients with T1DM.^18^ At the same time, current assays cannot distinguish between IAA and insulin antibody which is induced by insulin therapy, IAA has been excluded in some studies.^19^ Therefore, we did not focus on IAA in our study. Although there was no significant difference in the titer of diabetes‐associated autoantibodies (GADA, IA2A, and ZnT8A) between the two groups in our study, the titers of multiple autoantibodies were higher in children with DKA, and the positive rate of ZnT8A had a diagnostic value for the development of DKA. ZnT8A positivity was more frequent in the Finland Study Group, which was similar to our results.^20^ ZnT8A was not a single positive diabetic‐related autoantibody; it was associated with the positivity of IA2A and GADA. Moreover, the positivity of multiple diabetes‐related autoantibodies was significantly higher in ZnT8A‐positive subjects compared to ZnT8A‐negative subjects. Similarly, Lampsona et al observed that ZnT8A was mostly present together with GADA and IA2A, which was associated with a younger age of diagnosis. The research demonstrated that ZnT8A‐positive patients had a higher incidence of multiple diabetes‐related autoantibodies, which meant more severe β‐cell dysfunction and a higher prevalence of DKA.^21^ ZnT8 is a 369‐amino acid pancreas‐specific zinc transporter, which is encoded by the SLC30A8 gene at chromosome 8q14.11, and SLC30A8 is a major target of humoral autoimmunity in T1DM.^22^ ZnT8 plays a key role in glucose homeostasis. A study suggested that ZnT8 was a crucial protein for both zinc accumulation and regulation of insulin secretion in pancreatic β cells.^23^ Compared with other autoimmune markers, ZnT8A is highly β‐cell‐specific and ZnT8A expression may not occur until there is enough β‐cell damage. Therefore, ZnT8A had been used as an independent predictor of diabetes risk and had been included in risk prediction and prevention studies for T1DM with DKA.^15^

In our study, a higher level of HbA1c was exponentially related to the risk of DKA. HbA1c positivity predicted the occurrence of complications, and patients with HbA1c levels >9% were prone to DKA.^24^, ^25^ The half‐life of hemoglobin is 120 days, which can reflect the average blood glucose level of patients in 2–3 months. Therefore, patients with T1DM are regularly monitored for HbA1c; it is a comparatively easily accessible risk marker for DKA.^17^ Many studies had confirmed that a higher level of HbA1c is an independent risk factor for diabetes complications. The risk of nephropathy increased at HbA1c levels >7.0%.^26^ Chronic hyperglycemia is regarded as the main pathogenic factor of MA.^27^ Although a study confirmed a causal relationship between higher blood glucose levels in the development and progression of DN, the relative importance of other risk factors could not be fully addressed.^7^ In addition, high serum levels of inflammatory proteins have been associated with the presence of MA in T1DM patients. Inflammation, increased apoptosis, and abnormal activation of the endothelium are increasingly considered to be major mechanisms in the development of MA.^27^ Our study revealed that ZnT8A positivity was of importance for the incidence of MA in individuals with T1DM. Hence, we can infer that positive ZnT8A can accelerate the progression of T1DM, leading to complications and comorbidity. These findings may provide targeted clinical strategies for the frequency of screening, prevention, and treatment of DN in T1DM.

This study presented some limitations. Firstly, our research was a single‐center study. Therefore, we need to conduct multi‐center and multi‐regional collaborative researches and follow‐up. Secondly, we had a comparatively small sample size of patients; actually, this was a preliminary study to explore the role of autoantibodies. Thirdly, the definite relationship between transient MA levels and diabetic nephropathy deserves further study. A follow‐up study should be conducted to verify the findings. Fourthly, some autoantibodies, including ZnT8A, might have vanished by the time of the study in a proportion of patients. Regular measurement of the patient's autoantibodies during the disease may provide additional insight into the autoimmune process.

## 5 CONCLUSIONS

The positive ZnT8A had diagnostic value for DKA in children with T1DM and had the highest specificity among the three kinds of autoantibodies. Moreover, ZnT8A positivity was related to a higher titer of IA2A and more frequent occurrence of multiple diabetes‐related autoantibodies. Besides, the presence of positive ZnT8A was an independent risk factor of MA in children with T1DM. Therefore, we can infer that positive ZnT8A may be related to ketoacidosis and microalbuminuria, accelerating the progression of T1DM.

## CONFLICT OF INTEREST

The authors state no conflict of interest.

## AUTHOR CONTRIBUTIONS

M.Z., X.W., and R.W. are responsible for the work described in this paper. J.S., X.Z., and C.G. conceived, designed, and/or planned the study, and interpreted the results. X.W., R.W., and L.P. conducted data analysis. M.Z. and X.W. drafted the manuscript. C.C., W.Y., and L.L. critically reviewed and/or revised the manuscript for important intellectual content. All authors have accepted responsibility for the entire content of this manuscript and approved its submission.

## Data Availability

The data that support the findings of this study are not publicly available due to the privacy of research participants but are available from the corresponding author upon reasonable request.
